# Maternal PTSD and corresponding neural activity mediate effects of child exposure to violence on child PTSD symptoms

**DOI:** 10.1371/journal.pone.0181066

**Published:** 2017-08-02

**Authors:** Daniel S. Schechter, Dominik A. Moser, Tatjana Aue, Marianne Gex-Fabry, Virginie C. Pointet, Maria I. Cordero, Francesca Suardi, Aurelia Manini, Marylène Vital, Ana Sancho Rossignol, Molly Rothenberg, Alexandre G. Dayer, Francois Ansermet, Sandra Rusconi Serpa

**Affiliations:** 1 Child and Adolescent Psychiatry Service, University of Geneva Hospitals and Faculty of Medicine, Geneva, Switzerland; 2 Division of Developmental Neuroscience, Department of Psychiatry, Columbia University College of Physicians and Surgeons, New York, NY, United States of America; 3 Department of Psychiatry, Icahn School of Medicine at Mount Sinai, New York, NY, United States of America; 4 Division of Experimental Psychology and Neuropsychology, Department of Psychology, University of Bern, Bern, Switzerland; 5 Department of Mental Health and Psychiatry, University of Geneva Hospitals and Faculty of Medicine, Geneva, Switzerland; 6 Faculty of Health, Psychology and Social Care, Manchester Metropolitan University, Manchester, United Kingdom; Stellenbosch University, SOUTH AFRICA

## Abstract

The aim of this study was to examine the relationship of maternal interpersonal violence-related posttraumatic stress disorder (IPV-PTSD), associated neural activity in response to mother-child relational stimuli, and child psychopathology indicators at child ages 12–42 months and one year later. The study tested the hypothesis that decreased maternal neural activity in regions that subserve emotion regulation would be associated with child symptoms associated with emotional dysregulation at both time points. Functional magnetic resonance imaging of 42 mothers with or without violence-exposure and associated IPV-PTSD were assessed. Their child’s life-events and symptoms/behaviors indicative of high-risk subsequent PTSD diagnosis on a maternal-report questionnaire were measured one year later. Maternal IPV-PTSD severity was significantly associated with decreased ventromedial prefrontal cortex (vmPFC) activation in response to mother-child relational stimuli. Maternal IPV-PTSD severity and decreased vmPFC activation were then significantly associated with a child attachment disturbance at 12–42 months and symptoms/behaviors one year later, that were correlated with emotional dysregulation and risk for child PTSD. Maternal IPV-PTSD and child exposure to IPV were both predictive of child PTSD symptoms with maternal IPV-PTSD likely mediating the effects of child IPV exposure on child PTSD symptoms. These findings suggest that maternal IPV-PTSD severity and associated decreased vmPFC activity in response to mother-child relational stimuli are predictors of child psychopathology by age 12–42 months and one-year later. Significant findings in this paper may well be useful in understanding how maternal top-down cortico-limbic dysregulation promotes intergenerational transmission of IPV and related psychopathology and, thus should be targeted in treatment.

## Introduction

The relational model of posttraumatic stress disorder (PTSD) [1] supports the notion that the parent-child relationship can either buffer the child from risk or increase the risk of subsequent developmental psychopathology in the face of the child-parent exposure to violent trauma. Recently, Scheeringa and Zeanah [2] gave evidence for their model’s validity with empirical findings that maternal PTSD and co-morbid psychopathology mediate the effects of traumatic exposures on the development of PTSD in young children. Particularly salient as a predictor of child PTSD symptom development and severity was maternal self-report of avoidance as a coping strategy [2]. The authors of the present paper have similarly cited examples when traumatized mothers reported that when their infant or toddler was distressed, they would put on headphones and listen to music, go out into the hall to smoke, retreat into the bathroom, or put their child in his room and close the door when the child cried rather than approaching him, thereby demonstrating impairment in age-appropriate limit-setting [3, 4].

One longitudinal study showed that maternal history of abuse and neglect becomes predictive of less parenting sensitivity only once the children are above the age of 12 months and thus are more mobile and gesturally expressive [5]. This suggests evidence for a sensitive period during which the adverse impact of parental PTSD in the context of history of maltreatment and other interpersonal violence is most pronounced; and of which the timing may be later than that of other parental mental illness. Maternal major depression, for example, has been shown in one study to have a more important effect on maternal behavior than PTSD during the first months of life [6].

These findings largely support those of other research groups who focused their studies more specifically on the impact of maternal interpersonal violence-related PTSD on disturbances in the mother-child relationship in early childhood particularly around effects of maternal avoidance and emotional numbing on mutual emotion regulation beyond the first year of life [7]. Additional studies have demonstrated that child dysregulated aggression, and other externalizing behaviors and internalizing symptoms (i.e. anxiety) in families with domestic violence, were significantly associated with maternal interpersonal violence-related posttraumatic stress disorder (IPV-PTSD) severity, even after controlling for a child’s direct exposure to the violence [8, 9].

Studies have begun to identify patterns of maternal neural activity corresponding to maternal sensitivity or lack thereof in response to child stimuli involving the medial prefrontal and insular cortices, as well as the hippocampal and parahippocampal areas, thalamus and striatal sub cortical areas [10, 11]. Additional literature has focused on maternal neural activity in response to stressful versus non-stressful mother-child stimuli among mothers with IPV-PTSD [12–14]. In particular, IPV-PTSD in adult patients has long been associated with decreased activity in the medial prefrontal cortex (mPFC) in response to trauma-related stimuli [15, 16]. More recently, maternal IPV-PTSD has been associated with decreased mPFC activity in response to mother-child relational stimuli (i.e. mother-child separation versus play; adult men and women in menacing versus non menacing interactions as related to maternal behavior) [13, 17, 18]. Yet no study, to the authors’ knowledge, has yet examined the relationship of maternal IPV-PTSD-related neural activity to child symptoms.

An important consideration in the longitudinal examination of developmental psychopathology among infants and toddlers of IPV-PTSD affected mothers is the fact that a) the children's exposure to violence is often unknown and b) the mothers' unpredictable, non-contingent behavior (i.e. retreating in one instance of child distress and becoming hostile and controlling in another instance) and chronic emotional dysregulation (i.e. crying in front of the child in frustration with his behavior, smiling or laughing when the child is distressed, escalating when the child is distressed rather than calming the child) can be significantly associated with symptoms of an attachment disturbance that can overlap with child PTSD symptoms even in the absence of a clear traumatic event [19, 20]. Few studies have looked systematically at both forms of psychopathology: attachment disturbances and PTSD symptoms in pre-school age children of mothers with IPV-PTSD.

The form of attachment disturbance that commonly occurs in families at high risk for intergenerational occurrence of violence and maltreatment given parental traumatic history, phenomenologically falls neither into the traditional inhibited nor disinhibited subtypes of Reactive Attachment Disorder [21]. Lieberman and Zeanah [22], have described this atypical attachment disturbance as a "Secure Base Distortion" (SBD) subtype characterized by separation anxiety, auto-aggressive and otherwise self-endangering behavior, hypervigilance (i.e. "frozen watchfulness"), and role-reversal. The current study proposes to examine the relationship of maternal IPV-PTSD to neural activity in response to mother-child interactive stimuli and to SBD symptoms on the Disturbance of Attachment Interview (DAI) [23] as all of these variables might predict child PTSD symptoms one-year later.

We hypothesized the following:

1a) maternal IPV-PTSD severity will be associated with a disturbance of attachment characterized at 12–42 months of age (T1). 1b) Maternal IPV-PTSD at T1 will also be related to the severity of maternally reported child symptoms and behavior problems on a screening measure of child PTSD at 24–54 months of age, one year later (T2).
2) the two principal areas of the medial prefrontal cortex that are important to emotion regulation and extinction of the fear response (both vmPFC and dmPFC) [4, 24] will be less activated among mothers with greater IPV-PTSD symptoms in response to video excerpts of toddlers in a stressful condition inducing a helpless, distressed state. Less activation in these areas will also be associated with child psychopathology across two time points: SBD symptoms at T1 as well as greater severity of child behavior problems at T2.

## Methods

### Participants

The institutional ethics committee at the Geneva University Hospitals approved this research project which is in accordance with the Helsinki Declaration [25]. Women and their children were recruited by flyers posted at the university hospitals and faculties, as well as community centers, daycares, and schools. Domestic violence agencies were also included to oversample for violence-exposed mothers. All women who responded with interest were screened.

Inclusion and exclusion criteria were as follows: Biological mothers were included in the study if they had lived with their child for the majority of the child's life. Mothers were excluded if they were active substance-abusers or suffering from a psychotic disorder. Children aged 12–42 months were included. Mothers and children were excluded from the study if mothers were still breastfeeding, or physically and/or mentally impaired to an extent interfering with participation in laboratory tasks.

Seventy-two mothers and children were screened and provided informed consent. Four mothers were found to have a full PTSD-diagnosis or clinically significant symptoms (subthreshold) due to a non-IPV traumatic event (i.e. medical-surgical event, accident, natural disaster, etc.) and were thus excluded from the present analysis. There were 3 exclusions due to contra-indications to undergoing MRI (i.e. claustrophobia, implants), and 6, due to unusable MRI data (motion artifact, structural malformation). Of the remaining 59 participants, 26 met criteria for DSM-IV-TR PTSD diagnosis and 10 had clinically significant symptoms (PCLS score > 25 and CAPS score>30; PCLS = Post-traumatic Symptom Checklist-Short Version, CAPS = Clinician Administered PTSD Scale) that were below the threshold for full-diagnosis, but all of these mothers were included independent of whether their IPV-related trauma was primarily due to domestic violence, to violent encounters with non-intimates, or to childhood maltreatment. These 36 full- and subthreshold-diagnosis mothers were analyzed as one group given the comparable clinical needs of full- and subthreshold-diagnosis subjects who displayed distress and dysfunction due to PTSD symptoms. Twenty-three mothers were in the non-PTSD group. Out of the 59 dyads who had complete fMRI and behavioral data at baseline, we obtained 48 completed questionnaires one year later (n = 28 in the IPV-PTSD group; n = 20 in the non-PTSD group). Sixty-five percent of the non-PTSD control group consisted of women with French as their mother-tongue; whereas only 36% of women in the PTSD group consisted of women who named French as their maternal language.

## Procedure

A screening session was followed by two visits, T1a and T1b, separated by 1 to 2 weeks. This was followed by the MRI scan (T1c) approximately 1 to 3 weeks later. During the screening session, following informed written consent, mothers were given a socio-demographic and life-events interview. During T1a, mothers were interviewed without their child, with a focus on the mother's mental representations of her child, mother-child relationship, her traumatic life-events, and her psychopathology. During T1b, mothers brought their child for a parent-child laboratory procedure, followed by additional measures about the child. One year later (T2), mothers received a letter inviting them to complete attached questionnaires to inquire into their children's subsequent life-events, symptoms and behaviors. After each visit, mothers received 50 Swiss francs (approximately $50) along with a book or toy for their child. Interview guides for visits T1a and T1b can be found in the supplementary information.

### fMRI session

fMRI stimuli were drawn from mother-child interaction sequences of free-play and separation embedded within the 25-minute mother-child interaction (i.e. Modified Crowell Procedure) [26]. A research assistant who was blind to case-control status selected the silent excerpts for the fMRI stimulus of play and separation: Mothers viewed the play-excerpt that was rated most joyful and reciprocal, and the separation-excerpt rated most emotionally negative and distressed. Mothers viewed 6 silent, 30-second video-excerpts of 3 children, each during the two conditions (separation and play): 1) own child, 2) unfamiliar boy, and 3) unfamiliar girl. The unfamiliar children conditions were obtained by filming two non-participant mothers and their children for which mothers had given written informed consent for these videos to be used in the experiment and related presentations to professional audiences. The order of presentation was pseudo-randomized across both blocks and participants. The fMRI study design was described in a previous publication [12].

All data including maternal interview, parent-child observation and symptom scales at T1 were collected over a period of 4–6 weeks. T2 data involving maternal-report questionnaires were completed at a single time-point one-year after completion of the T1 study.

### Measures

Screening Session: We conducted an interview with the mothers using the Geneva Socio-demographic Questionnaire (GSQ) [27]. The family socio-economic status (SES) was calculated using the Largo Index [28]. Mothers completed the Conflicts Tactics Scale 2 Short Version (CTS) [29], which measured violence in the mother’s current or otherwise most recent romantic partnership.

T1a: Mothers completed the Geneva Child Exposure to Violence Questionnaire (CETV; [30]). The CETV is an adaptation for parents of the violence subscale of the Child Exposure to Domestic Violence Scale (CEDV; α = .78) [31]. This 10-item measure asks the mother about child exposure to domestic violence and investigates the frequency (4 points-scale from “never” to “almost always”) a child witnessed the violence (i.e. the child was next to his mother or saw/heard violence) and who was the main actor (i.e. father of the child, family member). Each event was given a score that increased with the number of instances and the closeness of the main actor (i.e. father scores higher than uncle and uncle higher than stranger). The CETV showed good convergent validity with the maternal Conflict Tactics Scale (r = .40, p < .005) and good internal consistency (α = .75). Of note, there were 8 missing values on the CETV due to this measure having being introduced after initial subjects were seen. Among those subjects who did not complete the measure, 7 were in the IPV-PTSD group. The authors note that other forms of child exposure to trauma (i.e. maltreatment, accidents, medical-surgical interventions etc) were probed using a standard life events checklist [32], but that for the purposes of testing our a-priori hypotheses and reducing the number of comparisons, only analyses involving the CETV measure are reported.

The Clinician Administered PTSD Scale (CAPS) was applied using the version compatible with the DSM-IV. A comparison between CAPS scores and the Structured Clinical Interview for DSM IV (SCID) interview demonstrated that the CAPS was a valid and reliable measure with high sensitivity (90%) and specificity (95%), and low misclassification rate (7.1%) concerning PTSD that is linked to interpersonal and community violence [33]. All CAPS items had Kappa coefficients higher than 0.63; internal consistency for all CAPS items resulted in a Cronbach's alpha coefficient of 0.97 [33]. Similarly, the Posttraumatic Symptom Checklist—Short Version (PCL-S) compatible with the DSM-IV demonstrates validity and high reliability[34]: Cronbach's alpha coefficients indicated high internal consistency for the total scale (0.91) and for re-experiencing, avoidance, and hyperarousal symptom clusters as delineated in the DSM-IV (0.83, 0.81, and 0.80). Temporal reliability (test-retest) was high and consistent for different cutoffs. Additionally, parental stress was measured via the Parenting Stress Index—Short Form (PSI-SF), which is a standardized 36-item parent-report measure that shows high internal consistency (Cronbach’s alpha 0.92) [35].

T1b: For the mother-child visit, mothers were asked to bring their child to the lab for a parent-child interaction procedure [26] followed by administration of measures focusing on the child's life-events, psychopathology, and social-emotional development. One of these measures, the DAI, was administered to mothers and completed by the clinician based on mothers' responses and clinical observations, as stipulated in the manual. Observations by the administering clinician of child behaviors pertinent to the interview items take priority in the clinician’s follow-up questioning and clinical judgment leading to coding of the interview items. The application of the DAI in the present study reflects this integration of clinician judgment and observation. The DAI measures 3 subscales: inhibited (min-max score 0–10), disinhibited (min-max score 0–6); and SBD (DAI-SBD, min-max score 0–8) with anchors. The SBD subscale included four behaviors that we hypothesized a-priori would be related to maternal IPV-PTSD severity: self-endangering behavior, separation anxiety, hypervigilance, and role-reversal. These four behaviors together attained a Cronbach’s alpha of 0.70.

T2: The follow-up invitation mailing sent one year after the baseline study included questionnaires to update socio-demographic data along with questions about traumatic exposures to mother and child that may have occurred during the intervening year, and the Child Behavior Checklist PTSD-Symptom Scale (CBCL-PTSD) in preschool age children. A subset of 20 items of the CBCL has been validated to serve as a screen for posttraumatic stress symptomatology. Dehon and Scheeringa [36] further adapted the measure for the CBCL 1.5–5 version using 15 items (minimum-maximum score 0–45). Our study included children ages 12–42 months who could not themselves verbally verify that their symptoms and behaviors were explicitly linked to a specific traumatic event. However, according to the CBCL-PTSD Scale when compared to children from non-PTSD mothers, children of IPV-PTSD mothers showed more symptoms and behaviors that are associated with risk for the development of later PTSD if exposed to family violence.

### Statistical analysis

Due to the possibility of maternal language being a confounder, we performed a control analysis for maternal mother tongue (French vs non-french). All partial correlations controlling for maternal mother tongue did not change significance status for any correlation, neither behavioral only or with MRI, nor did it significantly influence any regressions.

Due to the possibility of maternal history of drug use being a confounder, we performed a control analysis for maternal mother tongue (French vs non-french). All partial correlations controlling for prior maternal drug use did not change significance status for any correlation, neither behavioral only or with MRI, nor did it significantly influence any regressions.

### Behavioral data

The chi-square test and the Student's t-test were used to compare IPV-PTSD and non-PTSD groups. Associations were tested using Spearman’s rank correlation coefficients, with Bonferroni corrections when applicable. Partial correlation coefficients were used to test associations, while controlling for additional variables. Multiple linear regression models were used to investigate the relationship between child behavioral problems associated with risk for PTSD at T2 (outcome) and child exposure to violence (predictor), and the mediating effects of maternal PTSD severity and vmPFC activity. Statistics were computed using SPSS version 22 (IBM Corporation, Armonk, NY, USA). The PROCESS for SPSS software was used to test for mediation effects and estimate 95% confidence intervals with a bootstrap method [37]. All tests were two-tailed, with significance level at 0.05.

### fMRI data

In first-level fMRI analysis, we produced a contrast between the average neural activity in response to seeing separation among all children (i.e. own child, unfamiliar boy and girl; which were shown to mothers in a randomized, mixed sequence of stimuli) as compared to play. This means that we took the sum of the average neural activity from seeing unfamiliar children during separation and the average neural activity when mothers saw their own child during separation and from that sum we subtracted the sum of the average neural activities when mothers saw unfamiliar and own children during play. In 2^nd^-level analysis we applied Pearson correlations to compare this contrast with the DAI-SBD subscale-score at T1 within one whole-brain analysis, and with child behavioral problems on the CBCL PTSD Subscale from T2 in another whole-brain analysis.

## Results

**Fig 1:**
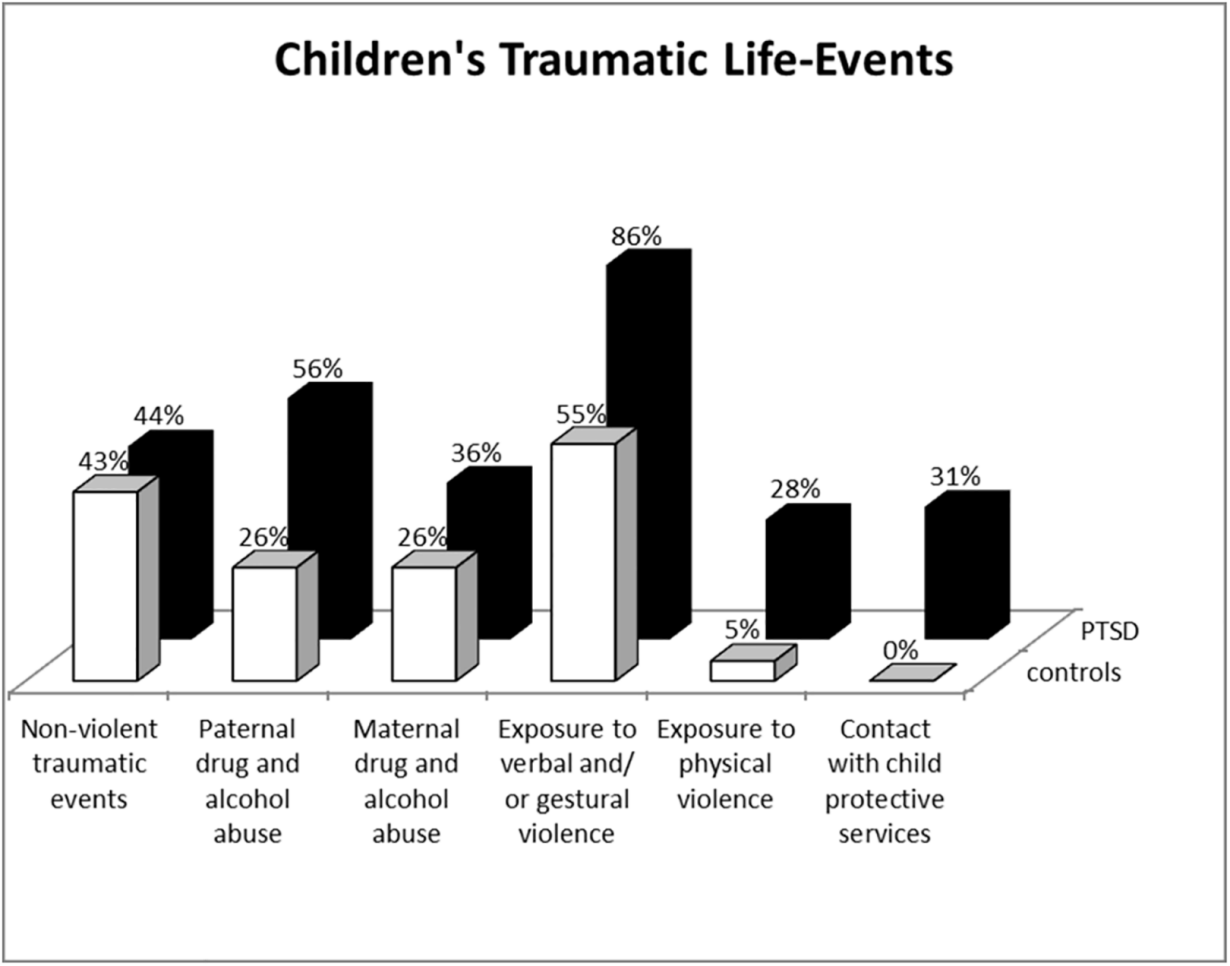
Distribution of children's exposure to violence by maternal report using the Child Exposure to Violence Questionnaire.

As expected, IPV-PTSD mothers and children differed from non-PTSD controls across a number of key variables (Table 1). Fig 1 shows the distribution of children's exposure to violence by maternal report.

**Table 1:** Characteristics of IPV-PTSD and non-PTSD mothers and children.

		All (n = 59)	Controls (n = 23)	IPV-PTSD (n = 36)	p-value
**Maternal Variables**	**Age of the mother in years**	34.2 (5.7)	34.9 (5.1)	33.7 (6.0)	.465
	**Socio-economic status (higher score means lower status)**	5.3 (2.2)	4.2 (2.0)	6.0 (2.1)	.002
	**Number of children the mother has**	1.8 (0.8)	1.8 (0.7)	1.8 (0.8)	.809
	**Native speaker of French**	44% (26/59)	65% (13/23)	36% (13/36)	.181
	**Domestic violence by partner in current/last partnership (score CTS)**	2.6 (6.3)	0.0 (0.0)	4.2 (7.7)	.002
	**Domestic violence by mother in current/last partnership (score CTS)**	1.0 (2.9)	0.2 (0.6)	1.5 (3.6)	.040
	**Maternal exposure to physical abuse as a child**	53% (31/59)	39% (9/23)	61% (22/36)	.103
	**Maternal exposure to sexual abuse as a child**	20%(11/56)	10% (2/21)	26% (9/35)	.099
	**Witnessed domestic violence as a child**	46% (26/56)	23% (5/22)	62% (21/34)	.003
	**Experienced domestic violence or other physical or sexual assault as an adult**	54% (32/59)	9% (2/23)	83% (30/36)	< .001
	**Experienced violence by stranger as an adult**	63% (37/59)	30% (7/23)	83% (30/36)	< .001
	**Experienced sexual assault as an adult**	15% (8/53)	0% (0/20)	24% (8/33)	.003
	**Non-violent experiences rated as "traumatic"**	40% (23/57)	26% (6/23)	50% (17/34)	.064
	**Parenting Stress Index (PSI)**	40.5 (21.5) ^1^	33.1 (15.9)	45.6 (23.4) ^1^	.031
	**History of prior drug use**	4%	4% (1/23)	22%(8/36)	.064
**Child variables**	**Age of the child in months**	27.5 (8.2)	27.1 (8.4)	27.7 (8.2)	.785
	**% Boys**	53% (31/59)	57% (13/23)	50% (18/36)	.943

% (n/number of valid data) and mean (SD) are reported.

^**1**^ There were two missing values within the IPV-PTSD group.

Table 2 shows correlations of maternal and child measures at T1 and T2. In accordance with our a priori hypotheses, maternal IPV-PTSD severity was associated with increased DAI -SBD at T1 (r = .39, p = .002) and severity of child behavior problems on the CBCL-PTSD Subscale at T2 (r = .38, p = .007). The DAI-SBD subscale at T1 correlated significantly with the CBCL PTSD subscale at T2 (r = .41, p = .004). Stratified analysis suggested that these associations were primarily driven by the IPV-PTSD group (Tables A and B in S1 File). Due to its importance as a potential explanation for our findings, we also looked at child exposure to violence at T1: it was significantly correlated to both the DAI-SBD (r = .31, p = .028) and the CBCL PTSD scale (r = .34, p = .026). We also tested whether these correlations were confounded by child age using partial correlation. Correlation coefficients and associated p-values remained virtually unchanged when controlling for child age (not shown).

**Table 2:** Correlation matrix of maternal and child measures at T1 and T2.

	Time	N^1^		DAI Secure Base Distortion	Child CBCL PTSD	Maternal PTSD (CAPS)	Child exposure to violence (CETV)
**DAI Secure Base Distortion**	T1	59	r =	1	** **	** **	** **
			p =				
**Child CBCL PTSD**	T2	48	r =	0.41	1		
			p =	0.004			
**Maternal PTSD (CAPS)**	T1	59	r =	0.39	0.38	1	
			p =	.002 ^#^	0.007		
**Child exposure to violence (CETV)**	T1	51	r =	0.31	0.34	0.53	1
			p =	0.028	0.026	< .001 ^#^	
**Partner Violence (CTS)**	T1	59	r =	0.48	0.27	0.37	0.41
			p =	< .001 ^#^	0.061	0.004	0.003
**Parenting Stress Index (PSI)**	T1	57	r =	0.3	0.46	0.38	0.23
			p =	0.025	.001 ^#^	0.003	0.104
**Socio economic status (higher score means lower status)**	T1	59	r =	0.29	0.26	0.44	0.38
			p =	0.027	0.084	.001 ^#^	0.007

^1^ There were 8 missing values on the CETV due to this measure being introduced after initial subjects were seen. Among those 8, 7 were in the IPV-PTSD group. There were 2 missing values on the PSI, all in the IPV-PTSD group.

^#^ Statistically significant after Bonferroni correction for 18 tests (p < .0028)

As indicated in Table 2, a number of correlations remained significant after Bonferroni correction for multiple tests. In particular, maternal IPV-PTSD and self-reported severity of partner violence during current or last intimate relationship, as measured by the CTS, remained significantly correlated with the DAI-SBD. CBCL-PTSD symptom severity in children at T2 was significantly associated with the Parenting Stress Index (PSI), but not with the child exposure to violence (CETV), the partner violence (CTS) or the severity of maternal IPV-PTSD.

**Fig 2:**
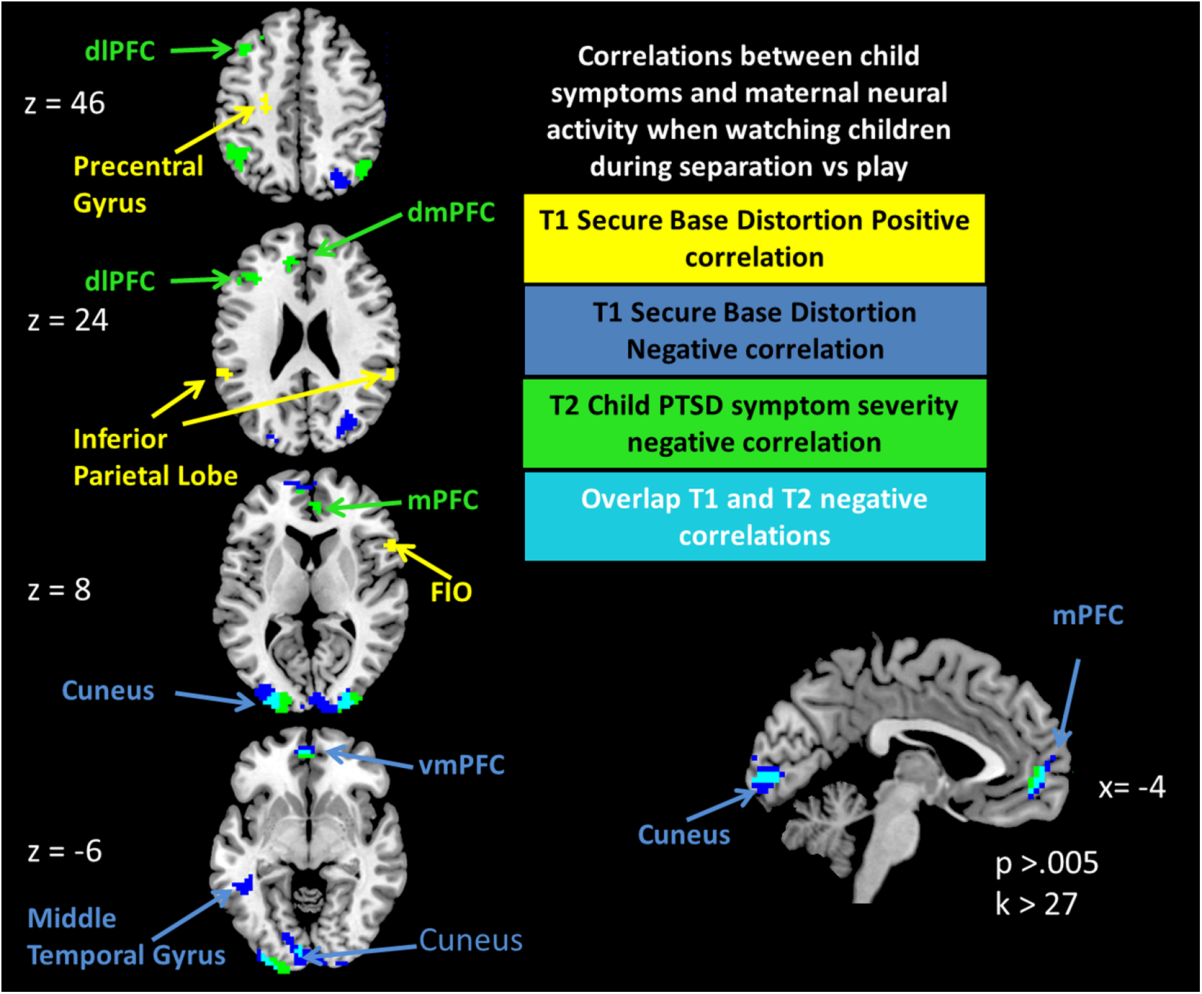
Correlation between child symptoms and maternal neural activity when watching children during separation vs. play. Abbreviations: mPFC = medial Prefrontal Cortex, dmPFC = dorsomedial Prefrontal Cortex, dlPFC = dorsolateral Prefrontal Cortex, vmPFC = ventromedial Prefrontal Cortex, FIO = Frontal Inferior Operculum.

fMRI results: In accordance with our hypotheses, the contrast of brain activity in response to children in separation vs. play in the vmPFC correlated negatively with the maternal IPV-PTSD, the DAI-SBD score at T1 and the child CBCL-PTSD score at T2 (Table 3 and Fig 2). Among other regions, activity in the dmPFC extending to the dorsal anterior cingulate cortex (dACC) correlated negatively with the child CBCL-PTSD score at T2, but not the maternal IPV-PTSD and DAI-SBD scores.

**Table 3:** Maternal brain activity when watching children during separation vs play.

Region	Cluster size	x	y	z	Peak voxel of the overall correlation with the population	Correlation of the entire cluster			
						Group	T1 DAI Secure Base Distortion (n = 59)	T1 Lifetime maternal IPV-PTSD (n = 59)	T2 Child CBCL PTSD (n = 48)
**Clusters with significant negative correlations of BOLD with the DAI SBD**									
Ventromedial Prefrontal Cortex	66	0	56	-2	t = 3.97	All	r = -.45	r = -.37	r = -.36
					p < .001		p < .001	p = .004	p = .012
						IPV-PTSD	r = -.46	r = -.31	r = -.38
							p = .004	p = .062	p = .044
						Control	r = -.15	r = .02	r = -.05
							p = .503	p = .940	p = .834
Right Superior Occipital Lobe	200	33	-76	25	t = 4.10	All	r = -.45	r = -.35	r = -.18
					p < .001		p < .001	p = .006	p = .224
						IPV-PTSD	r = -.41	r = -.25	r = -.12
Also Included: Right Middle Occipital Lobe							p = .014	p = .147	p = .556
						Control	r = -.37	r = -.07	r = -.02
							p = .083	p = .743	p = .942
Left Middle Temporal Gyrus	59	-45	-46	-5	t = 4.09	All	r = -.44	r = -.24	r = -.37
					p < .001		p < .001	p = .068	p = .011
						IPV-PTSD	r = -.51	r = -.13	r = -.38
							p = .002	p = .435	p = .048
						Control	r = -.11	r = .17	r = -.13
							p = .611	p = .445	p = .580
Cuneus / V1	350	6	-100	4	t = 5.17	All	r = -.546	r = -.378	r = -.418
					p < .001		p < .001	p = .003	p = .003
						IPV-PTSD	r = -.61	r = -.27	r = -.45
							p < .001	p = .106	p = .016
						Control	r = -.12	r = .21	r = -.14
							p = .569	p = .339	p = .548
Left Cuneus / V1	222	-24	-91	19	t = 4.83	All	r = -.52	r = -.44	r = -.45
					p < .001		p < .001	p = .001	p = .001
						IPV-PTSD	r = -.61	r = -.38	r = -.50
Also Included: Left Middle Occipital Gyrus							p < .001	p = .023	p = .007
						Control	r = .02	r = .21	r = -.05
							p = .913	p = .347	p = .830
**Clusters with significant negative correlations of BOLD with the CBCL PTSD**									
Ventral Anterior Cingulate Cortex	65	-3	53	-2	t = 3.43	All	r = -.30	r = -.23	r = -.48
					p = 0.001		p = .020	p = .080	p = .001
						IPV-PTSD	r = -.33	r = -.25	r = -.63
Also included: Ventromedial Prefrontal Cortex							p = .047	p = .148	p < .001
						Control	r = -.11	r = .09	r = -.19
							p = .609	p = .683	p = .422
Dorsomedial Prefrontal Cortex	46	-12	41	25	t = 4.18	All	r = -.17	r = -.20	r = -.48
					p <0.001		p = .208	p = .120	p = .001
						IPV-PTSD	r = -.18	r = -.31	r = -.54
also included: Dorsal Anterior Cingulate Cortex							p = .305	p = .063	p = .003
						Control	r = -.04	r = .03	r = -.27
							p = .843	p = .877	p = .245
Left Dorso- lateral Prefrontal Cortex	118	-42	20	37	t = 3.60	All	r = -.18	r = -.342	r = -.55
					p <0.001		p = .176	p = .008	p < .001
						IPV-PTSD	r = -.26	r = -.46	r = -.59
							p = .131	p = .005	p = .001
						Control	r = -.16	r = .06	r = -.00
							p = .478	p = 771	p = .991
Left Inferior Parietal Lobule	101	-45	-52	46	t = 3.60	All	r = -.16	r = -.32	r = -.45
					p <0.001		p = .211	p = .013	p = .001
						IPV-PTSD	r = -.15	r = -.32	r = -.43
							p = .368	p = .054	p = .021
						Control	r = -.00	r = -.03	r = -.33
							p = .982	p = .883	p = .149
Right Middle Occipital Gyrus / V1	91	21	-100	16	t = 4.02	All	r = -.42	r = -.33	r = -.49
					p <0.001		p = .001	p = .010	p < .001
						IPV-PTSD	r = -.48	r = -.19	r = -.50
							p = .003	p = .267	p = .007
						Control	r = -.06	r = .30	r = -.29
							p = .791	p = .158	p = .213
Left Middle Occipital Gyrus / V1	267	-24	-97	13	t = 4.47	All	r = -.45	r = -.39	r = -.53
					p <0.001		p < .001	p = .002	p < .001
						IPV-PTSD	r = -.56	r = -.27	r = -.62
Also included: Cuneus							p < .001	p = .106	p < .001
						Control	r = -.17	r = .17	r = -.10
							p = .447	p = .421	p = .662
Right Inferior Parietal Lobe	76	48	-64	43	t = 4.51	All	r = -.214	r = -.40	r = -.50
					p <0.001		p = .104	p = .001	p < .001
						IPV-PTSD	r = -.24	r = -.40	r = -.43
							p = .16	p = .014	p = .022
						Control	r = .10	r = -.02	r = -.41
							p = .647	p = .923	p = .067
Cerebellum	96	18	-82	-20	t = 3.70	All	r = -.31	r = -.34	r = -.46
					p <0.001		p = .016	p = .008	p = .001
						IPV-PTSD	r = -.34	r = -.09	r = -.48
							p = .040	p = .600	p = .009
						Control	r = .32	r = .25	r = -.01
							p = .133	p = .256	p = .951

The contrast of maternal brain activity in response to separation vs. play also correlated negatively with child CBCL-PTSD scores in the ventral anterior cingulate cortex (vACC), the left dorsolateral prefrontal cortex (dlPFC) and several non-frontal regions (Table 3). We also used partial correlations to test whether the effects of any of these clusters were confounded by child age or maternal SES. All clusters still correlated at r>.37 and p < .05 when controlling for these possible confounding factors.

Regression models: We performed multiple linear regression to test if maternal IPV-PTSD mediates effects of child exposure to violence (CETV) on child CBCL-PTSD at T2 among the 42 dyads with a completed CETV questionnaire. The CETV correlated with maternal IPV-PTSD severity (Table 2) and each of these two variables independently predicted the T2 CBCL-PTSD score (IPV-PTSD β = .38, p = 007; CETV β = .34, p = .026). Together in one model, each variable lost significance (R^2^ = .19, F(2,39) = 4.53, variance inflation factor = 1.56, p = .017; CETV β = .14, p = .438; IPV-PTSD β = .34, p = .070). A bootstrap test for mediation was significant for maternal IPV-PTSD likely mediating effects of CETV on child PTSD symptoms (indirect effect = .0184, 95% CI .0031-.0398).

We used a similar model to test if maternal vmPFC activity alone would possibly mediate the effects of CETV on child behavior problems on the CBCL-PTSD subscale. CETV correlated with maternal vmPFC activity as a biomarker of maternal PTSD (r = -.32, p = .022). Each of these two variables independently predicted the CBCL-PTSD scale one year later at T2 (vmPFC β = -.43, p = .002; CETV β = .34, p = .026). Together in one model, only vmPFC activity remained a significant predictor of child behavior problems on the CBCL-PTSD subscale (R^2^ = .22, F(2,39) = 5.46, p = .008, variance inflation factor = 1.39; CETV β = .22, p = .151; vmPFC β = -.34, p = .031). Maternal vmPFC activity significantly mediated the effect of child exposure to violence on child symptomatology associated with risk for PTSD at T2 (indirect effect = .0111, 95% CI .0003-.0272). When testing the same models within each group, neither group showed a significant mediation effect; this is possibly due to a loss of statistical power.

## Discussion

Upon testing our first hypothesis, we found a) that maternal IPV-PTSD severity was significantly associated with the disturbance of attachment characterized as a “secure base distortion” (SBD) at T1 and b) that maternal IPV-PTSD severity was significantly associated with the severity of child symptoms and behaviors on the CBCL-PTSD subscale indicative of significant risk for PTSD at T2. This finding is particularly important because we know that the majority of children of IPV-PTSD affected mothers’ experienced family violence, making the risk for PTSD among the children by T2 much higher. That being said, the limited verbal capacity of most children at ages 24–36 months is such that we prefer to say that these children are at significant risk for PTSD since not all are able to confirm that their symptoms are linked to their specific violent life-events. The degree of maternally reported CETV also predicted these at-risk child symptoms/behaviors. In support of the relational PTSD model [1, 3], we found that maternal IPV-PTSD may mediate the effects of CETV on subsequent child symptoms/behaviors on the CBCL-PTSD Subscale. The present study also echoes previous findings from studies of high-risk samples in which maternal IPV-PTSD predicted child dysregulated aggression, avoidance and hypervigilance on story-stem completion, as well as internalizing and externalizing symptoms although none of those previous studies examined the relationship between maternal neural activity as correlated with maternal IPV-PTSD and child symptoms [8, 9, 38].

This study further extends the existing literature in that it shows the relationship of maternal neural activity in response to stressful parent-child film-stimuli and maternal IPV-PTSD symptoms, secure base distortion, and child behavior problems associated with risk for PTSD one year later. More specifically, we previously found that maternal neural activity of the medial prefrontal cortex (mPFC) was a marker of maternal IPV-PTSD in both the ventromedial (vmPFC) and dorsomedial (dmPFC) regions [13]. Importantly, in the current study neural activity in this same region, the vmPFC was also negatively and significantly correlated with elevated scores on the T1 DAI-SBD subscale and on the T2 severity of child symptoms and behavior problems reported by mothers on the CBCL-PTSD subscale.

Neural activity in the vmPFC and dmPFC, together with the ventral anterior cingulate cortex (vACC), has been shown to be involved in the regulation and extinction of the limbic fear response in previous studies [39]. Thus, lower activation of the mPFC in response to the separation-play paradigm could be a useful indicator of maternal emotion dysregulation in research studies of intervention involving a similarly violence exposed population of mothers and preschool-age children. An important question remains: Can an effective parent-child intervention increase neural activity in top-down regulatory brain-areas such as the ventral medial prefrontal cortex? Previous studies support that the separation vs. play stimuli used in the present study were potent triggers of trauma-associated helpless states of mind that were mirrored by decreased activity in the mPFC, in proportion to the severity of the mothers’ IPV-PTSD [12, 13]. The least we can say is that maternal IPV-PTSD and related mPFC activity share a common variance that is predictive of later child behavior problems. Psychotherapeutic intervention that result in increased mother-child regulation of emotion, arousal, and behavior, we hypothesize would show effects in the mPFC.

This study was, however, not able to discern if reduced mPFC activity in response to stressors would be an etiologic factor in the development of maternal IPV-PTSD or whether it remains a simple biological correlate. We have noted a convergence of findings suggesting that the lower neural activity in the mPFC—and vmPFC in particular—corresponds to parents who have emotional regulation difficulties [40]. These emotional regulation difficulties are mirrored by cortico-limbic dysregulation within the maternal brain [13, 18].

Particularly novel is that for the first time, to our knowledge, decreased maternal neural activity in response to mother-child relational stimuli (i.e. mother-child separation versus play) in the mPFC and most saliently the vmPFC, holds potential predictive value for child developmental psychopathology. The vmPFC is one brain area that is essential to emotion regulation and is hyporeactive in mothers to seeing their own and unfamiliar children of a similar age, in stressed and helpless states of mind (i.e. during separation) vs. non-stressed states during which the child expresses agency (i.e during play) on silent video clips in the MRI scanner.

Supported by previous studies [41] we speculate that IPV-PTSD mothers, who have difficulty in appraising their child’s emotional communication, perceive their relationship with their child during stressful moments differently than non-PTSD mothers. We have previously described that mothers with IPV-PTSD may defensively interpret separation anxiety and clinging behavior, as anger and controlling behavior, and become frightened and avoidant of the child at the very moment the child is in need of his attachment figure [42, 43].

Limitations of this study include a sample size that did not permit more detailed analysis such as path analysis or to consider the possibility that associations between maternal and child psychopathology were due more or less to shared genetic vulnerability. We tested mediation only of the effects of child exposure to IPV by maternal IPV-PTSD on child symptoms and behaviors given our a-priori hypothesis. While mothers and young children are involved in a bidirectional or mutual system of emotion regulation, we have in this paper considered that mothers have many more capacities to comprehend, modulate and respond to their toddler’s emotional communication. Nevertheless, it is also possible, but beyond the scope of this paper to test, the child psychopathology could also mediate traumatogenic effects of maternal experience of the child’s behavior or other external stressors on maternal PTSD symptoms. An additional limitation is that we relied on maternal report for child exposure to violence and symptom follow-up at 1 year, due to the fact that mothers and children were contacted by mail and telephone rather than being asked to come in person. As stated, children below the age of 36-months cannot reliably confirm that their symptoms follow from their experience of a traumatic event even though observational data might strongly suggest that this is the case. Additionally, when testing for mediation, the analysis involved a measure of child exposure to violence solely dependent on maternal report, the latter, which could have been subject to the mother’s minimization or exaggeration. Finally, as is consistent with the literature [8, 44], IPV-PTSD affected mothers had lower SES than non-PTSD mothers. Due to the significant correlation between IPV-PTSD severity and SES we cannot completely dissociate the influences of SES and IPV-PTSD given the limitations of this study. It is thus possible that SES is a confounding or mediating factor of the effects of IPV-PTSD on maternal behavior. And it is similarly possible that the distress and dysfunction implicit to chronic IPV-PTSD can impair the capacity to obtain or sustain gainful employment and pre-requisite training [45]. One further limitation is that ethnic origin was not matched across the groups and could possibly be a confounder in the observed associations.

## Conclusion

We have demonstrated that maternal IPV-PTSD is a possible predictor of child psychopathology and of child symptoms and behaviors associated with risk for PTSD even after controlling for child direct exposure to family violence, which among children of mothers with IPV-PTSD was noted to occur in over 85% of cases [1, 2]. Moreover, we have found a potentially specific maternal indicator (i.e. decreased mPFC activity in the separation-play fMRI paradigm) for mother-toddler relational disturbance and subsequent child psychopathology that may be useful for examining treatment effects on affected mother-child dyads. The paper lends all the more weight to the importance of addressing family violence and actively treating parental PTSD as early as possible in order to interrupt intergenerational cycles of IPV and related psychopathology.

## Supporting information

S1 File Supplementary tables including correlations of Questionnaire, and MRI data, split by group. (PDF)

S2 File Deidentified SPSS database including the relevant data for this Study. Furthermore this file Brain imaging contains contrast images, containing the clusters that were significantly correlated with the Disturbance of Attachment Interview at T1 and the Child Behavior Check List at T2: these files can be visualized using a number of freely available programs, including MRICron.
(ZIP)

S3 File Interview Guide used for visit T1A. (PDF)

S4 File Interview Guide used for visit T1B. (PDF)
