# Supplementary material for: Maternal PTSD and corresponding neural activity mediate effects of child exposure to violence on child PTSD symptoms
Source: PLoS One. 2017 Aug 2;12(8):e0181066. doi: 10.1371/journal.pone.0181066 (PMC5540394; doi:10.1371/journal.pone.0181066)
Supplement: S1 File — (PDF) [file pone.0181066.s001.pdf]

# Supplementary tables including correlations of Questionnaire, and MRI data, split by group.

**S1 Table A: Correlation matrix among non IPV-PTSD controls only**

| | N | | DAI Secure Base Distortion | CBCL PTSD | Maternal vmPFC activity | Maternal PTSD (CAPS) | Child exposure to violence |
|---|---|---|---|---|---|---|---|
| DAI Secure Base Distortion | 23 | r =<br>p = | 1 | | | | |
| CBCL PTSD | 20 | r =<br>p = | .179<br>.451 | 1 | | | |
| Maternal vmPFC activity | 23 | r =<br>p = | -.135<br>.538 | -.081<br>.735 | 1 | | |
| Maternal PTSD (CAPS) | 23 | r =<br>p = | -.062<br>.780 | -.004<br>.986 | -.060<br>.785 | 1 | |
| Child exposure to violence | 22 | r =<br>p = | -.287<br>.196 | -.255<br>.277 | -.439<br>.041 | -.234<br>.296 | 1 |
| Parenting Stress Index | 23 | r =<br>p = | .255<br>.240 | .369<br>.110 | -.233<br>.284 | .081<br>.712 | .157<br>.485 |
| Partner Violence (CTS) | 23 | r =<br>p = | No Control | Reported | any partner | violence | |
| Socio economic status (low values better) | 23 | r =<br>p = | -.157<br>.457 | .003<br>.991 | .217<br>.319 | .501<br>.015 | -.260<br>.242 |

**S1 Table B: Correlation matrix among IPV-PTSD mothers only**

| | N | | DAI Secure Base Distortion | CBCL PTSD | Maternal vmPFC activity | Maternal PTSD (CAPS) | Child exposure to violence |
|---|---|---|---|---|---|---|---|
| DAI Secure Base Distortion | 36 | r =<br>p = | 1 | | | | |
| CBCL PTSD | 28 | r =<br>p = | .400<br>.035 | 1 | | | |
| Maternal vmPFC activity | 36 | r =<br>p = | -.469<br>.004 | -.527<br>.004 | 1 | | |
| Maternal PTSD (CAPS) | 36 | r =<br>p = | .271<br>.110 | .250<br>.199 | -.291<br>.085 | 1 | |
| Child exposure to violence | 29 | r =<br>p = | .359<br>.056 | .336<br>.127 | -.151<br>.435 | .296<br>.119 | 1 |
| Parenting Stress Index | 34 | r =<br>p = | .219<br>.214 | .419<br>.033 | -.463<br>.006 | .338<br>.051 | .076<br>.705 |
| Partner Violence (CTS) | 36 | r =<br>p = | .484<br>.003 | .200<br>.308 | -.099<br>.565 | .183<br>.285 | .338<br>.073 |
| Socio economic status (low values better) | 36 | r =<br>p = | .328<br>.058 | .206<br>.313 | -.136<br>.443 | .145<br>.414 | .446<br>.020 |
