# Supplementary material for: Maternal PTSD and corresponding neural activity mediate effects of child exposure to violence on child PTSD symptoms
Source: PLoS One. 2017 Aug 2;12(8):e0181066. doi: 10.1371/journal.pone.0181066 (PMC5540394; doi:10.1371/journal.pone.0181066)
Supplement: S3 File — (PDF) [file pone.0181066.s003.pdf]

Projet d'Interaction entre Mère et Enfant
Visite 1 – Protocole

**Durée**	**CM: ______**
5 min	Expliquer la procédure :<br><br>1. Questionnaire et Entretien avec mère<br>2. La visite entière sera enregistrée<br><br>**Consigne -** Introduction aux questionnaires en début d'entretien :<br>***…Pendant l'entretien nous allons aborder trois domaines de votre vie:***<br><br>*Dans la première partie je vais vous poser des questions qui portent sur votre* ***relation avec votre enfant (WMCI)***<br><br>*Dans la deuxième partie des questions qui portent sur votre* ***réaction aux événements stressants que vous avez vécu dans votre vie et dont on a déjà parlé la dernière fois.***<br><br>***Ensuite on verra l'heure qu'il est, il y a trois questionnaires que vous pouvez aussi remplir à la maison et amener au prochain rdv***<br>(note : on peut aussi proposer un autre rendez-vous pour terminer les questionnaires de ce rendez-vous)<br><br>***Et dans la troisième partie on parlera de comment vous avez vécu votre grossesse.***<br><br>***1) Dans cette première partie je vais vous poser des questions qui portent sur votre relation avec votre enfant***<br>Première partie: « Working Model of the Child Interview »<br><br>Remarque: laisser parler la mère, respecter les questions du WMCI, ne pas trop développer. Attention au temps (45 min optimal)<br><br>2)**…**Je vais parler avec vous de vos réactions aux événements de vie stressants que vous avez vécus (CAPS).<br>Demander à la mère de choisir les trois évènements les plus stressants qu'elle a pu vivre dans sa vie, et reprendre selon ce qui a été abordé dans le rendez-vous préalable (éligibilité). Choix de l'évènement le plus stressant pour CAPS et PCL-S(PCLS : Je vais vous demander de vous référer à la manière dont vous vous êtes sentie au cours du dernier mois).<br><br>3) …On va maintenant parler de la grossesse (questionnaire stress prénatal)

2 heures	Entretien avec la mère :<br>Semi-structuré<br>___WMCI (ne pas ajouter de questions, laisser parler la dame).<br>___CAPS (x2 si + ev. Traumatiques)<br>___PCL-S<br>___STRESS PRENATAL<br>___BDI<br>___Hopkins
	Questionnaires pour la mère (possible de les remplir à domicile)<br>___ALEX<br>___PSI
