# Supplementary material for: Maternal PTSD and corresponding neural activity mediate effects of child exposure to violence on child PTSD symptoms
Source: PLoS One. 2017 Aug 2;12(8):e0181066. doi: 10.1371/journal.pone.0181066 (PMC5540394; doi:10.1371/journal.pone.0181066)
Supplement: S4 File — (PDF) [file pone.0181066.s004.pdf]

MODE EMPLOI RENDEZ-VOUS 2

Durée	Tâche/Mesure<br>Nom Primaire : ______
15 min	**Mode emploi Rdv 2**<br><br>**Avant le rdv : regarder plages horaires pour IRM avec Dominik**<br>(Madame arrive avec la feuille remplie du jour même).<br><br>***Accueil, consigne générale pour le rdv et ensuite*** - *réception feuille avec les informations sur les activités et horaires du jour même pour la mère et l'enfant*<br><br>Expliquer la procédure :<br><br>1. Cortisol mère et enfant<br>2. Jeux/Activités et Séparation/Réunion, puis quelque chose d'inhabituel (collègue déguisé, puis jouet étrange)<br><br>3. Questionnaire et Entretien avec mère (assistant de recherche jouera avec l'enfant)<br>(4. Cadeau emballé) (possibilité de l'expliquer après avoir terminer toute la procédure des interactions mère-enfant)<br>5. La cassette enregistrera la visite entière<br><br>**CONSIGNE POUR LA SEQUENCE D'INTERACTIONS**<br><br>***Nous allons vous proposer de jouer avec votre enfant assis à cette petite table . Vous jouerez avec lui durant quelques minutes et je frapperai à la porte : ce sera pour vous le signe de sortir de la pièce. Votre enfant restera dans la pièce, il sera vu par les caméras en régie. Je vous donnerai un chronomètre et ce qu'on vous demande c'est de mesurer le temps et d'attendre 3 minutes derrière la porte dans le couloir, je ne serais pas loin.***<br><br>***Ensuite, après les 3 minutes, vous rentrerez retrouver votre enfant.<br>Je vous donnerai un autre matériel de jeu, vous jouerez de nouveau avec votre enfant et puis je frapperai de nouveau à la porte et vous re-sortirez pendant 3 minutes.<br>Quand vous rentrerez, vous jouerez à nouveau et puis quelque chose d'un peu inhabituel va se passer : Un collègue entrera dans la pièce un peu déguisé, puis lorsqu'il re-sortira, c'est moi-même qui rentrerai dans la salle avec un jouet un peu inhabituel.***<br><br>***Ensuite, cette séquence sera terminée, normalement 30 minutes se seront écoulées et ce sera le moment de refaire un prélèvement de salive pour vous et votre enfant.***<br><br>~~***Autre chose qu'on va regarder aujourd'hui : toutes les 30 minutes une collègue va venir pour vous et votre enfant, pour le prélèvement de salive.***~~<br><br>**Pour la séquence d'interactions, SE RAPPELER :**<br><br>1. Consigne: Ne pas dire à la mère qu'il y a un clown.<br><br>• **Si la mère demande ce qu'elle doit dire à l'enfant en partant, lui répondre « Faites comme vous feriez habituellement».**<br><br>• **Si la dame demande si l'enfant sera seul, lui dire que « l'enfant**

	**est vu en régie au moyen des caméras pendant que vous êtes dehors. »**<br><br>2. Jeu libre : selon l'âge de enfant, poser la corbeille de jouets par terre (enfant d'âge_______)<br><br>3. Et, si enfant plus grand, petite table au milieu de la salle et la corbeille au milieu entre la mère et l'enfant, posée sur une chaise afin que ça puisse être filmé correctement)<br><br>Séparation: **Ne pas parler avec la mère pendant la séparation** (altération niveaux stress spontané et régulation de la mère). Dire quelque chose comme par exemple : « je ne suis pas loin » et se mettre vers les escaliers (ne pas rester trop proche de la mère).<br><br>4. Pendant la séparation, possible de parler avec la mère pour « est-ce que ça va pour vous? » -<br>« Voudriez-vous entrer ? » (On préfère que la mère rentre plutôt que la calmer. Ne pas interférer avec son niveau de stress en la rassurant.)<br><br>5. Si la mère demande d'entrer, on peut lui dire «Je sais que ce n'est pas facile ce qu'on vous demande, est-ce que vous pouvez tenir encore un peu ? ». Si c'est trop difficile, dire à la mère « Alors faites comme vous le pensez».<br><br>6. La mère ne doit pas parler à l'enfant derrière la porte. Si elle présente une trop grande détresse, lui dire qu'elle peut rentrer si elle pense que cela est préférable, mais éviter de parler à l'enfant derrière la porte.<br><br>7. Si la mère parle avec le case manager durant le moment de séparation, répondre très brièvement si question à propos de la situation. Mais si questions à propos d'autres choses, répondre « Je pourrais vous répondre après ».<br><br>8. Réunion: donner la consigne à la mère de frapper à la porte avant d'entrer.<br><br>**9. JEU : Demander de ranger les jouets du jeu libre avant de donner la consigne et les jouets du jeu structuré.**<br><br>10. En sortant, enlever de la salle la valise des jouets du jeu libre.<br><br>11. Objets nouveaux : **Dinosaure**<br><br>• Entrer dans la pièce avec le dinosaure caché sous le linge.<br>***Consigne à l'enfant* : « Je vais te présenter un copain, tu verras il est un peu bizarre mais il est très sympa ».** Poser le dinosaure sur la table face à l'enfant (la queue posée sur la table), le faire avancer et rugir. Durée de la présentation du dinosaure à l'enfant : env 3 min.<br><br>• **Ne pas rassurer l'enfant en présentant les jouets**
	• Lorsque la procédure du dinosaure est terminée, ranger le dinosaure sous le linge

	• Expliquer à la mère que cette première partie d'entretien est terminée et lui demander si tout va bien pour elle. Lui expliquer qu'un prochain échantillon de salive va être pris et que la suite du rendez-vous avec les entretiens et questionnaires suivra.<br><br>• Expliquer à la mère le débriefing clown : « En général, ce qu'on propose aux mamans c'est que le collègue qui est venu déguisé en clown revienne sans son déguisement pour montrer à l'enfant qu'il s'agit bien d'un déguisement. Souhaiteriez-vous qu'il revienne montrer son déguisement à la fin du rendez-vous ? »<br><br>• Expliquer la procédure du cadeau emballé : «Une dernière chose que l'on propose à l'enfant après avoir terminé la partie questionnaire c'est un cadeau emballé pour lequel on va lui demander d'attendre avant de l'ouvrir que je rentre dans la pièce. A mon retour il pourra l'ouvrir et l'emporter avec lui à la maison. On vous demande juste de ne pas interagir avec lui durant ce moment, (je peux vous donner des questionnaires à remplir pendant ce temps). »<br><br>Remarque : On peut proposer de l'eau à la mère et à l'enfant uniquement à la suite de la prise de salive, et retirer les verres après- ils ne doivent pas boire durant les 30 prochaines minutes avant le prochain échantillon de salive.
½ heure<br>(+ 3min après pour cortisol)	**Heure commencé :** ___________<br>**8 min** – Jeux : ___<br>**3 min** – Séparation 1 ___<br>**2 min** – Retrouvailles/Jeux ___<br>**2 min** – Ranger ___<br>**4 min** – Activité Structurée ___<br>**3 min** – Séparation 2 ___<br>**2 min** – Retrouvailles ___<br>**3 min** – Clown ___<br>**3 min** – Jouet dinosaure ___
1h<br>+ 3 min après pour le cortisol	Entretien avec la mère (30 min)<br>___ DAI<br>___ Descripteurs<br>____ENFANT TEMOIN VIOLENCE<br>___ PCLS<br>___SCID<br>___ASQ<br>___ITSEA<br>___CADEAU EMBALLE<br>(___débriefing clown)<br><br>**(Peuvent être rempli à domicile) :**<br>___ITSEA<br>___ASQ<br><br>Pour le Cadeau emballé :<br><br>- La personne – case manager-entre avec un cadeau emballé au moment où Maribel a fini le prélèvement de cortisol (FIN PROTOCOLE CORTISOL)<br><br>- Faire asseoir l'enfant à la table. Ne pas montrer à l'enfant le cadeau avant qu'il soit assis.<br><br>- Procédure :<br><br>- Si l'enfant est en train de jouer, il faut poser le cadeau emballé devant l'enfant

	et lui dire :<br><br>**Consignes à l'enfant : « *Je vais te donner un cadeau mais tu dois attendre avant de l'ouvrir. Je vais sortir de la salle et tu ne dois pas toucher le cadeau pendant ce temps. Montre-moi que tu peux attendre.* »** Répéter la consigner un maximum de 2 fois.<br><br>Sortir de la salle pendant 2 minutes et revenir en disant à l'enfant : ***« Bravo ! Voilà je suis de retour, maintenant tu peux ouvrir ton cadeau. »***<br><br>~~Si l'enfant joue avec quelqu'un c'est cette personne qui amène le cadeau.~~<br><br>(Alternative à évaluer : la personne qui va jouer avec l'enfant pendant que la mère remplit les questionnaires amène dans la salle le cadeau emballé avant de jouer avec lui)<br><br>Débriefing Clown (si la mère le souhaite) et prise RDV IRM

	Argent donné ? Oui/Non
	Livre/jouet donné? Oui/Non
	Reçu signé? Oui/Non

Notes sur la visite entière

____

____

____

____

____

Prochaine visite prévue – Date : __________ Heure : __________
